# Oncogenic K-ras Induces Mitochondrial OPA3 Expression to Promote Energy Metabolism in Pancreatic Cancer Cells

**DOI:** 10.3390/cancers12010065

**Published:** 2019-12-25

**Authors:** Ning Meng, Christophe Glorieux, Yanyu Zhang, Liyun Liang, Peiting Zeng, Wenhua Lu, Peng Huang

**Affiliations:** 1 Sun Yat-sen University Cancer Center, State Key Laboratory of Oncology in South China, Collaborative Innovation Center for Cancer Medicine, Guangzhou 510060, China; mengning@sysucc.org.cn (N.M.); zhangyany@sysucc.org.cn (Y.Z.); liangly@sysucc.org.cn (L.L.); zengpt@sysucc.org.cn (P.Z.); luwenh@sysucc.org.cn (W.L.); 2 Metabolic Innovation Center, Sun Yat-sen University, Guangzhou 510080, China

**Keywords:** K-ras, mitochondria, OPA3, pancreatic cancer, energy metabolism

## Abstract

K-ras (Kirsten ras GTPase) mutations are oncogenic events frequently observed in many cancer types especially in pancreatic cancer. Although mitochondrial dysfunction has been associated with K-ras mutation, the molecular mechanisms by which K-ras impacts mitochondria and maintains metabolic homeostasis are not fully understood. In this study, we used two K-ras inducible cell systems, human pancreatic epithelial/ K-ras^G12D^ (HPNE/K-ras^G12D^) and human embryonic kidney cells with tetracycline repressorT-Rex/K-ras^G12V^, to evaluate the role of oncogenic K-ras in regulating mitochondrial function. Among a panel of genes known to affect mitochondria, only the expression of OPA3 (optic atrophy protein 3) was consistently up-regulated by K-ras activation in both cell lines. Importantly, high expression of OPA3 was also observed in clinical pancreatic cancer tissues. Genetic knockdown of OPA3 caused a significant decrease of energy metabolism, manifested by a suppression of oxygen consumption rate (OCR) and a decrease in cellular ATP content, leading to inhibition of cell proliferation capacity and reduced expression of epithelial–mesenchymal transition (EMT) markers. Our study suggests that OPA3 may promote cellular energy metabolism and its up-regulation in K-ras-driven cancer is likely a mechanism to offset the negative impact of K-ras on mitochondria to maintain energy homeostasis. As such, OPA3 could be a potential target to kill cancer cells with K-ras mutations.

## 1. Introduction

Mutations in *KRAS* gene are cancer-driven genetic events that are often seen in pancreatic ductal adenocarcinoma (90%) [1,2], lung [3], and colon [4] cancers (30–40%). The presence of mutated K-ras (Kirsten ras GTPase) in cancer cells is associated with a poor prognosis of the cancer patients due in part to the activation of several signaling pathways (e.g., nuclear factor kappa-B(NF-κB) and mitogen-activated protein kinase (MAPK)) by K-ras to enhance cell proliferation, migration, and invasion [5,6]. Mitochondrial dysfunction and metabolic alterations may also contribute to the highly malignant phenotype of K-ras-driven cells.

We have previously shown that K-ras (G12V) activation led to mitochondrial dysfunction, manifested by a decrease in oxygen consumption and an increase in generation of reactive oxygen species (ROS) [7,8]. By using a proteomic approach, we have further identified that down-regulation of NDUFAF1 (NADH dehydrogenase 1 alpha subcomplex assembly factor 1) might be responsible for the decreased mitochondrial respiration [9]. Indeed, activation of K-ras could induce a decrease in NDUFAF1 expression leading to mitochondrial dysfunction and an increased generation of ROS. It remains unclear, however, how the K-ras-driven cancer cells with mitochondrial dysfunction could maintain their homeostasis in energy metabolism.

Recently, K-ras activation has also been also correlated with dynamic changes of mitochondrial morphology and subsequent changes in cellular metabolism [10,11,12]. The morphology changes include alterations in mitochondrial fusion and fission. Biogenesis, mitophagy, and cell death are the other processes related to mitochondrial dysfunction [13]. The mitofusins (Mfn1 and Mfn2) are the main drivers of mitochondrial fusion whereas Drp-1 (dynamin-related protein 1) regulates mitochondrial fission [13]. Opa3 (optic atrophy protein 3) is another molecule proposed to affect the mitochondrial fission process. This protein is mutated in patients with hereditary optic neuropathies [14]. Opa3 is located at the outer membrane of the mitochondria, and has been shown to induce mitochondrial fission and could sensitize cells to pro-apoptotic agents such as staurospaurin [14]. However, the underlying mechanisms largely remain elusive. It is also unclear if OPA3 could affect mitochondrial respiratory function and cellular metabolism.

In this study, we used two cell models with inducible mutant K-ras to investigate the K-ras-induced molecules that might be involved in regulation of mitochondrial functions. Among many genes that might potentially modulate mitochondrial homeostasis, we identified OPA3 as a K-ras target gene that significantly impacts mitochondrial function and cellular energy metabolism.

## 2. Results

### 2.1. Oncogenic K-ras Activation Induces OPA3 Expression in HPNE/K-ras^G12D^ and T-Rex/K-ras^G12V^ Cells

**Figure 1:**
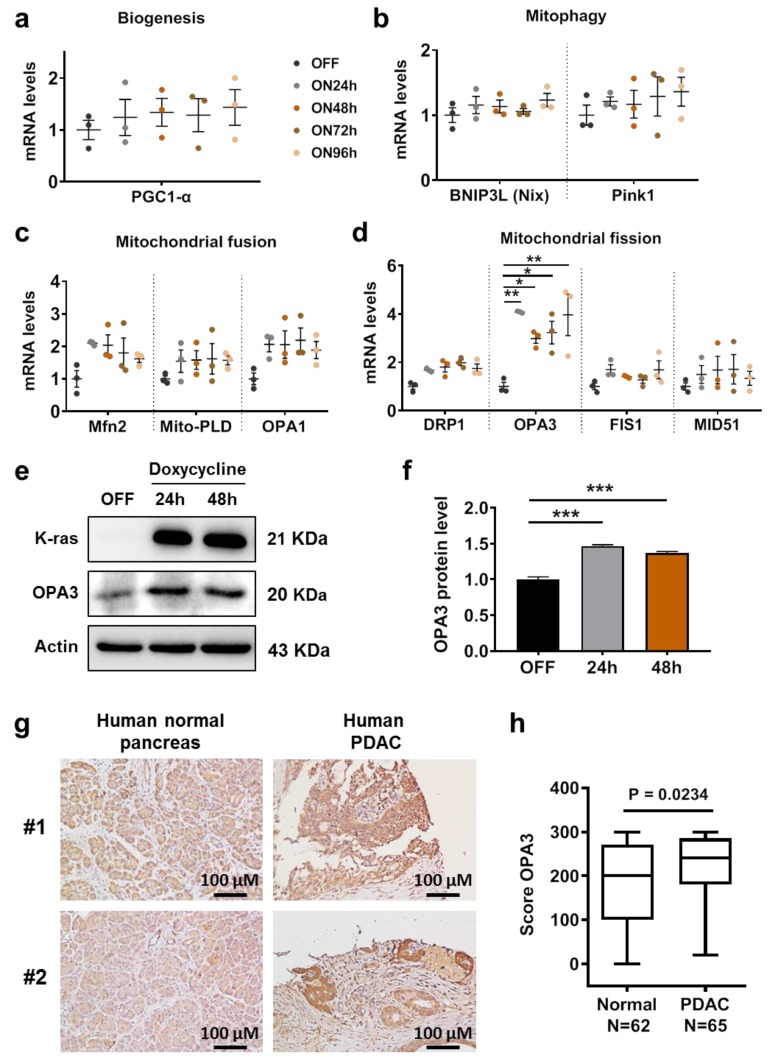
Oncogenic K-ras activation induces OPA3 expression in HPNE cells. Inducible K-ras^G12D^ cell line was incubated with doxycycline for various incubation times. The mRNA levels of (**a**) biogenesis, (**b**) mitophagy, (**c**) mitochondrial fusion, and (**d**) mitochondrial fission genes were measured by real-time PCR. (**e**,**f**) Inducible K-ras^G12D^ cell line was incubated with doxycycline for various incubation times. OPA3 protein levels were analyzed by immunoblotting and quantified. (**g**) Immunohistochemistry analysis for OPA3 in human normal and pancreatic (PDAC) tumor tissues. (**h**) Score of OPA3 staining, measured by immunohistochemistry IHC, in human normal and PDAC tumor tissues. Statistics: data are mean ± S.E.M, one-way ANOVA followed by Dunnett post hoc test (compared to “OFF”) for (**a**–**d**) and (**f**), and unpaired T-test for (**h**). * *p* < 0.05, ** *p* < 0.01, and *** *p* < 0.001. Abbreviations: BNIP3L (Nix): BCL2 interacting protein 3 like; Drp1: dynamin related protein 1; Fis1: mitochondrial fission 1 protein; Mfn2: mitofusin-2; HPNE: human pancreatic nestin expressing cells; K-ras: Kirsten RAS GTPase; MID51: mitochondrial dynamics proteins of 51 kDa; Mito-PLD: mitochondrial phospholipase D; OPA1: optic atrophy protein 1; OPA3: optic atrophy protein 3; PDAC: pancreatic ductal adenocarcinoma; PGC-1α: peroxisome proliferator-activated receptor gamma coactivator 1-alpha; and Pink1: PTEN induced kinase 1.

Our previous study showed that activation of K-ras led to mitochondrial dysfunction [7,9], we first used two doxycycline-inducible K-ras expression cell models, HPNE/K-ras^G12D^ and T-Rex/K-ras^G12V^, to test the potential effect of oncogenic K-ras on the expression of candidate genes involved in regulation of mitochondrial function and morphology. The candidate genes included PGC1-α(involved in regulation of mitochondrial biogenesis), BCL2 interacting protein 3 like (BNIP3L) and PTEN induced kinase 1 (Pink1) (involved in mitophagy), mitofusin 2 (Mfn2), mitochondrial phospholipase D (Mito-PLD), and optic atrophy protein 1 (OPA1) (involved in mitochondrial fusion); and dynamin related protein 1 (DRP1), OPA3, mitochondrial fission 1 protein (FIS1), and mitochondrial dynamics proteins of 51 kB (MID51) (involved in mitochondrial fission). Among all the genes tested, only OPA3 mRNA expression showed a consistent increase after K-ras was induced in human pancreatic epithelial HPNE/K-ras^G12D^ cells (Figure 1a–d), suggesting that OPA3 might be an important molecule downstream of K-ras that mediates the impact on mitochondria. Western blot analysis further confirmed the increase of OPA3 protein after K-ras^G12D^ induction (Figure 1e,f). Importantly, immunohistochemical staining revealed that OPA3 protein was highly expressed in clinical tumor specimens of pancreatic ductal adenocarcinoma (PDAC) as compared to human normal pancreatic tissues (Figure 1g,h). It is worth noting that in normal pancreas OPA3 appeared mainly in the acinar cells, whereas in PDAC tissues OPA3 was highly expressed in the tumor cells (Figure 1g).

The induction of OPA3 expression by K-ras was also observed in another K-ras-inducible cell line (T-Rex/K-ras^G12V^), which originated from human embryonic kidney cells [7]. As shown in Supplementary Figure S1, OPA3 mRNA was induced after K-ras^G12V^ was activated in a time-dependent manner. Interestingly, there was a moderate decrease in PGC1-α mRNA expression after K-ras^G12V^ induction in T-Rex/K-ras^G12V^ cells (Figure S1a), which was not observed in HPNE/K-ras^G12D^ cells (Figure 1a). The expression of another fission molecule DRP1 mRNA did not show consistent change after K-ras induction in the two cell lines (Figure 1d, Figure S1c). Western blot analysis revealed that K-ras activation mainly induced the phosphorylation of DRP1 protein in both cell lines (Supplementary Figure S2a–d), consistent with observations reported previously [11,15,16].

These data together demonstrated that K-ras activation mainly induced OPA3 expression in cell culture and in human pancreatic cancer tissues.

### 2.2. Impact of K-ras and OPA3 on Mitochondrial Morphology in Cancer Cells

**Figure 2:**
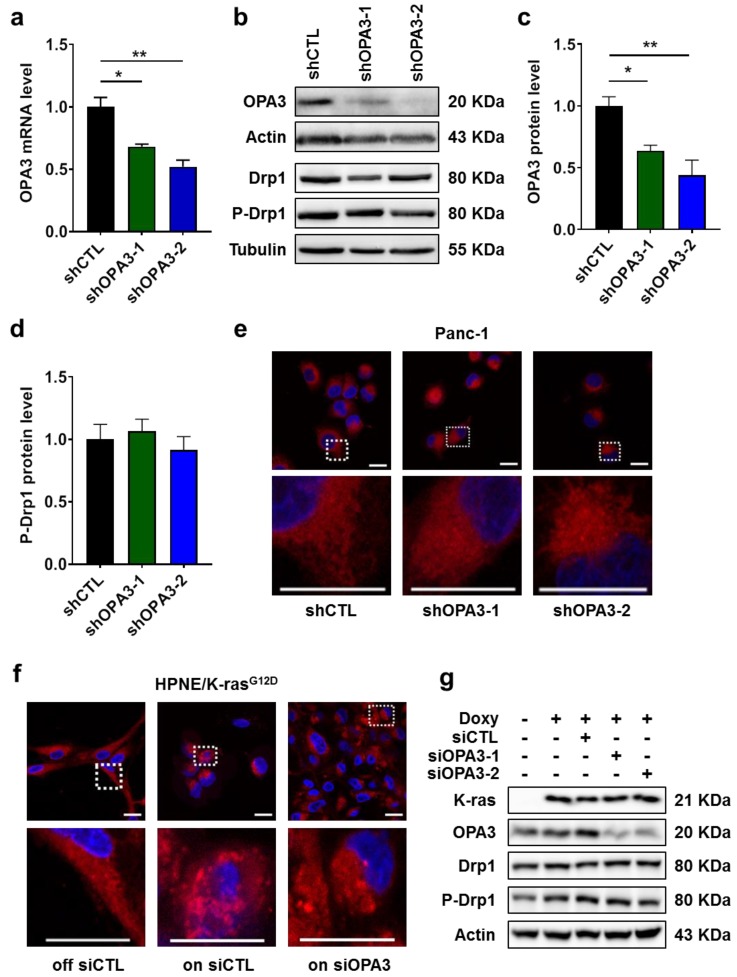
Silencing of OPA3 expression does not modify mitochondrial morphology: (**a**) OPA3 mRNA levels in Panc-1 OPA3-shRNA cell lines were measured by real-time PCR. (**b**–**d**) OPA3 and phospho-Drp1 (ser616) protein levels in Panc-1 OPA3-shRNA cell lines were analyzed by immunoblotting and quantified. (**e**,**f**) Mitochondrial morphology was analyzed with MitoTracker red probe in Panc-1 OPA3-shRNA cells and HPNE/K-ras^G12D^ cells (with or without doxycycline and siRNA against OPA3). Scale bars represent 20 μm (**g**) HPNE/ K-ras^G12D^ cell line was transfected with control siRNA (siCTL) or OPA3 for 48 h and then incubated with doxycycline for additional 48 h. K-ras, OPA3, Drp1, and phospho-Drp1 (ser616) protein levels were analyzed by immunoblotting. Statistics: data are mean ± S.E.M. (n = 3); one-way ANOVA followed by Dunnett post hoc test (compared to “shCTL”) for (**a**) and (**c**,**d**). * *p* < 0.05 and ** *p* < 0.01. Abbreviations: Drp1: dynamin related protein 1, K-ras: Kirsten RAS GTPase, OPA3: optic atrophy protein 3, shCTL: control shRNA, shOPA3: shRNA against human OPA3, siCTL: control siRNA, and siOPA3: siRNA against human OPA3.

Currently there is very little information on the biological function of OPA3 in cancer cells. To evaluate the role of OPA3 in K-ras-driven cancer cells, we first used shRNA strategy to construct a stable OPA3-knockdown cell line from Panc-1 cells, a human pancreatic cell line harboring K-ras^G12D^ mutation. The knockdown efficacy was accessed by qRT-PCR and immunoblotting, which showed that OPA3 mRNA and protein were reduced by 30–50% and 40–60%, respectively (Figure 2a–c). Based on the previous report that OPA3 might induce mitochondrial fragmentation [14], we quantified P-Drp1 expression (reflects activation state) and used MitoTracker to analyze the potential effect of OPA3 knockdown on mitochondrial morphology and intracellular distribution. Drp1 was phosphorylated and remained high in the three cell lines (Figure 2b,d). Consistent with Drp1 phosphorylation, the mitochondria in Panc-1 cells containing control shRNA (shCTL) exhibited mainly fragmented morphology, suggesting that mitochondria in the K-ras mutant cells are in a fission-dominant stage (Figure 2e). Interestingly, shRNA knockdown of OPA3 in Panc-1 cells (shRNA-OPA3) did not cause any significant change in mitochondrial morphology (Figure 2e). In HPNE/K-ras^G12D^ cells without doxycycline (K-ras OFF), mitochondria appeared elongated (Figure 2f, left panel), suggesting that the mitochondria in cells without K-ras activation were largely in the fusion stage. When K-ras was induced by adding doxycycline (Figure 2g), a large portion of cells exhibited fragmented mitochondria (Figure 2f, middle panel). Consistently, siRNA knockdown of OPA3 in K-ras/ON cells (Figure 2g) did not change the fragmented mitochondrial morphology (Figure 2f, right panel). These observations together suggest that activation of oncogenic K-ras could induce mitochondrial fragmentation (fission), likely due in part to induction of Drp1 phosphorylation (Figure 2g, Figure S2), whereas knockdown of OPA3 did not affect mitochondrial morphology in K-ras-driven cells.

### 2.3. Silencing of OPA3 Expression Suppresses Mitochondrial Respiratory Function in Pancreatic Cancer Cells

**Figure 3:**
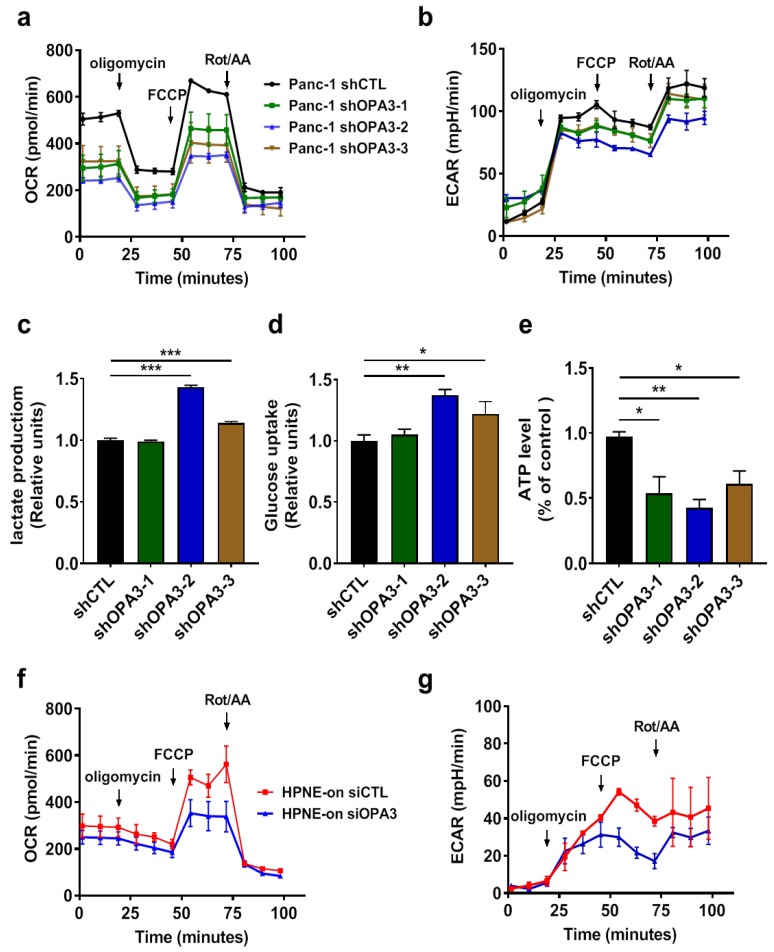
Silencing of OPA3 expression alters mitochondrial function in Panc-1 cells: (**a**,**b**) Real-time measurement of OCR and ECAR for Panc-1 cells using the Seahorse analyzer. (**c**,**d**) Lactate and glucose contents were quantified in culture medium of Panc-1 OPA3-shRNA cells. (**e**) Intracellular ATP content was quantified in Panc-1 OPA3-shRNA cells. (**f**,**g**) Real-time measurement of OCR and ECAR for HPNE/K-ras^G12D^ cells using the Seahorse analyzer. HPNE/ K-ras^G12D^ cell line was transfected with control siRNA (siCTL) or OPA3-siRNA for 24 h and then incubated with doxycycline for additional 24 h. Statistics: data are mean ± S.E.M. (n = 3); one-way ANOVA followed by Dunnett post hoc test (compared to “shCTL”) for (**c**–**e**). * *p* < 0.05, ** *p* < 0.01 and *** *p* < 0.001. Abbreviations: AA: antimycin A; ECAR: extracellular acidification rate; FCCP: carbonyl cyanide p-trifluoromethoxyphenylhydrazone; OCR: oxygen consumption rate; OPA3: optic atrophy protein 3; Rot: rotenone; shCTL: control shRNA; shOPA3: shRNA against human OPA3; siCTL: control siRNA; and siOPA3: siRNA against human OPA3.

Since shRNA knockdown of OPA3 did not cause any significant change in mitochondrial morphology, we further tested if OPA3 could affect mitochondrial function and cellular metabolism, using a Seahorse cellular metabolic analyzer to measure oxygen consumption rate (OCR) and extracellular acidification rate (ECAR) as the key indicators of mitochondrial oxidative phosphorylation and cytosolic glycolysis, respectively. As shown in Figure 3a, a knockdown of OPA3 by shRNA induced the significant decrease (approximately 50%) in basal OCR, suggesting that OPA3 could functionally promote mitochondrial respiration. Interestingly, the mitochondrial maximum respiration reserve capacity (revealed by adding FCCP) was also attenuated by OPA3 knockdown, and such a phenomenon was consistently observed in three shRNA-OPA3 cell lines (Figure 3a). Analysis of ECAR revealed no significant compensatory increase in glycolysis in the OPA3-knockdown cells (Figure 3b), although two of the shRNA-OPA3 clones (clones #2 and #3) exhibited a modest increase in glucose uptake and lactate production (Figure 3c,d). Measurement of cellular ATP showed a significant depletion of ATP in all OPA3-knockdown cell lines (Figure 3e), indicating that the slight increase in glycolysis was not sufficient to compensate the suppression of mitochondrial respiration in term of ATP generation. Consistent with the findings in Panc-1 cells, basal OCR levels and maximum respiration reserve capacity decreased in HPNE/K-ras/On cells with OPA3 silencing (Figure 3f). No compensatory increase in glycolysis was observed in this cell line (Figure 3g). These data together demonstrate that the up-regulation of OPA3 in K-ras-driven cancer cells might play a significant role in maintaining mitochondrial energy metabolism.

### 2.4. OPA3 Promotes Proliferation and Epithelial–Mesenchymal Transition in Panc-1 Cancer Cells

**Figure 4:**
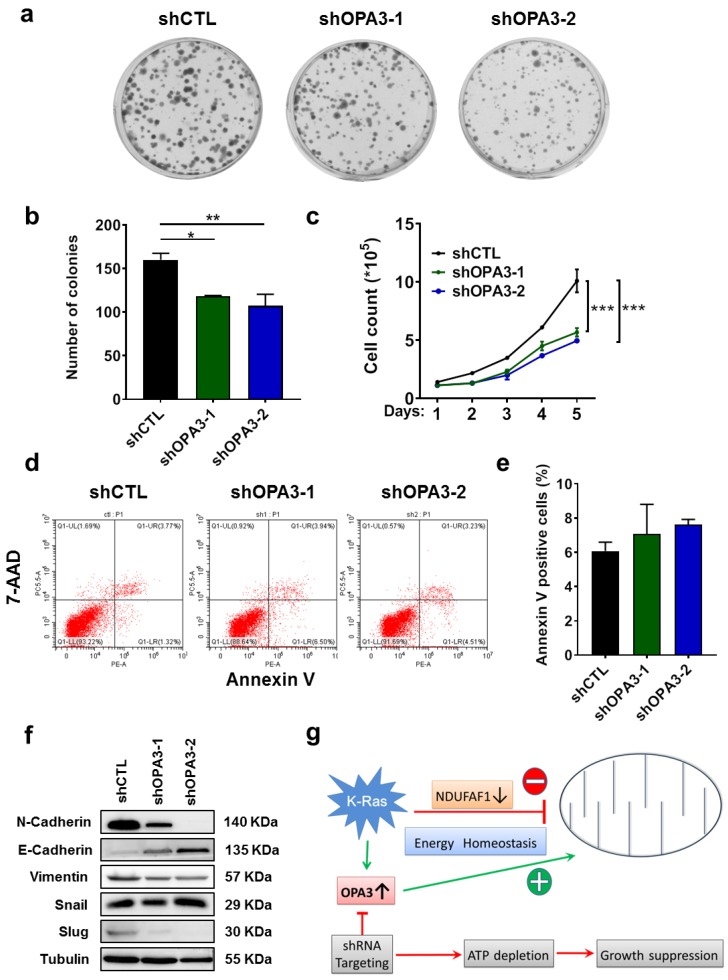
Silencing of OPA3 expression decreases cell proliferation and epithelial–mesenchymal transition (EMT): (**a**,**b**) Cell proliferation in Panc-1 OPA3-shRNA cells was analyzed by clonogenic assay and quantified. (**c**) Cell proliferation in Panc-1 OPA3-shRNA cells was analyzed by cell counting. (**d**,**e**) Apoptosis in Panc-1 OPA-shRNA cells was analyzed by flow cytometry and quantified by Annexin-V positivity. (**f**) Expressions of EMT-related proteins in Panc-1 OPA3-shRNA cells were analyzed by immunoblotting. (**g**) Representative scheme depicting the role of OPA3 expression induced by oncogenic signaling in pancreatic cancer cells. Statistics: data are mean ± S.E.M. (n = 3); one-way ANOVA followed by Dunnett post hoc test (compared to “shCTL”) for (**b**) and (**e**) and two-way ANOVA for (**c**). * *p* < 0.05, ** *p* < 0.01, and *** *p* < 0.001. Abbreviations: 7-AAD: 7-aminoactinomycin D, K-ras: Kirsten RAS GTPase, NDUFAF1: NADH dehydrogenase 1 alpha subcomplex assembly factor 1, OPA3: optic atrophy protein 3, shCTL: control shRNA, and shOPA3: shRNA against human OPA3.

Since metabolic changes might control signals that regulate basic cellular processes such as cell proliferation [17], we then tested if OPA3 might play a role in cancer cell proliferation, using both colony formation assay and direct cell counting. As shown in Figure 4a,b, clonogenic assay showed that silencing of OPA3 led to a 30% reduction of proliferation in Panc-1 cells. Consistent with these findings, direct cell counting reveal a significant decrease in cell proliferation in shRNA-OPA3 Panc-1 cell lines (Figure 4c). This decrease in cell counts was not due to an increase in cell death, since there was not significant increase of Annexin-V-positive cells after OPA3 knockdown (Figure 4d,e). Western blotting analysis was then used to examine the potential molecular events associated with the changes in cell proliferation. Interestingly, we observed a gene expression pattern consistent with a decrease in epithelial–mesenchymal transition (EMT) when OPA3 was knocked down, as evidenced by a decrease in expression of N-cadherin, vimentin, and slug, and an increased in E-cadherin expression (Figure 4f). These data together suggest that a main role of OPA3 in K-ras-driven cancer is to promote mitochondrial ATP generation to support cell proliferation.

## 3. Discussion

Mitochondria are important organelles that provide energy and metabolic intermediates for many biochemical pathways to support cell survival and proliferation. Mitochondrial dysfunction is often observed in cancer and contributes to tumorigenesis [18]. Oncogenic K-ras mutations are frequently detected in cancers. Although the presence of mutated K-ras is associated with mitochondrial dysfunction [7], the molecular mechanisms that regulate the changes in mitochondrial function and morphology still remain to be investigated. In this context, the oncogenic signaling has been shown to decrease complex I activity [9,19], enhance glycolytic activity [7,20], promote generation of ROS [7], and induce mitophagy [21]. Consistent with the previous studies, we demonstrated that K-ras could induce mitochondrial fission by activation of DRP1 via promoting its phosphorylation. This is in line with the finding that K-ras signaling could induce the phosphorylation of DRP1 through MAPK (mitogen-activated protein kinase) pathway [11,15,16].

The most significant new finding of our study is the discovery that K-ras could induce the expression of OPA3. Previous study showed that K-ras could induce mitochondrial dysfunction due to in part by a down-regulation of NDUFAF1, leading to elevated glycolysis [7]. However, it is unknown how the K-ras-driven cancer cells could maintain its homeostasis in mitochondrial energy metabolism with down-regulated NDUFAF1 that compromises the mitochondrial respiratory chain function. The current study showed that K-ras could also induce OPA3 expression, which seems to play a major role in promoting mitochondrial respiratory function to maintain homeostasis in ATP generation, since the knockdown of OPA3 led to significant decrease in OCR and cellular ATP. Based on these observations, we postulate that K-ras may on the one hand impose a negative impact on mitochondria through down-regulation of NDUFAF1, but on the other hand exert a positive impact on mitochondria respiration via up-regulation of OPA3 (Figure 4g). Such seemingly opposite regulatory mechanisms might ensure an overall metabolic homeostasis with high glycolysis in conjunction with functional mitochondrial metabolism to support the active proliferation of the K-ras-driven cancer cells, which requires both ATP and metabolite intermediates from glycolysis and the TCA cycle. Thus at the present time, it is unclear how OPA3 promotes mitochondrial respiratory activity in the presence of NDUFAF1 down-regulation. One possibility is that OPA3 promotes complex II activity, and thus bypasses the compromised complex I due to low NDUFAF1 expression. Another potential mechanism could involve alleviation of mitochondrial ROS stress by OPA3, and thus relieve the suppressive effect on mitochondria by ROS. These possibilities required further investigation in future studies.

Currently there is very limited information on the regulation of Opa3 expression and its biological function in cancer cells. One study showed that overexpression of wild-type Opa3 could induce mitochondrial fragmentation whereas a knockdown resulted in elongated mitochondria in HeLa and Cos7 cells [14]. Another study reported that TGF-β (transforming growth factor-beta) could induce loss of Opa3 which in turn caused elongation of mitochondria in retinal pigment epithelial ARPE-19 cells [22]. Our study suggested that in K-ras-driven cancer cells, OPA3 seems to mainly affect mitochondrial energy metabolism, and its impact on mitochondrial morphology appears minimal. The mechanism by which K-ras regulates OPA3 expression and its potential role in mitochondrial fission in cancer cells are other important areas for future study. It would also be important to know how OPA3 is regulated in vivo during the oncogenic K-ras-driven carcinogenesis process. One way to investigate this in vivo regulation would be to use established tumor mouse models such as KPC mice to examine the expression of OPA3 mRNA and protein in pancreatic tumor tissues at different stages of tumor development, and seek correlation between these molecular events with K-ras expression.

## 4. Materials and Methods

### 4.1. Cell Culture

The doxycycline inducible T-Rex/K-ras^G12V^ cells were constructed as previously described [7] and cultured in Dulbecco’s modified Eagle’s medium (DMEM) supplemented with 10% tetracycline-free fetal bovine serum (FBS). The doxycycline inducible HPNE/K-ras^G12V^ cells were cultured in 35% DMEM, 35% F-12K medium, and 20% M3 Base medium and supplemented with 10% tetracycline-free fetal bovine serum (FBS). Panc-1 cells (#CRL-1469) were from ATCC (Manassas, VA, USA) and cultured in DMEM supplemented with 10% tetracycline-free fetal bovine serum (FBS). All cell lines were confirmed to be mycoplasma negative (LookOut mycoplasma PCR detection kit; Sigma, Saint-Louis, MO, USA) and authentication of cell lines was performed by STR genotyping (Microread Genetics, Beijing, China).

### 4.2. Construction of shRNA Stable Cell Lines

Plasmid constructs psi-LVRU6GP containing shRNA against human OPA3 were from Genecoepia (Rockville, MD, USA). The sequences of the shRNA are: 5′-GCTTCGCGCCGTAGTCTTA-3′ (shCTL), 5′-GCGAGTTCTTCAAGACCTATA-3′ (shOPA3-1), 5′-GCGAGGGCATCATCTTCATCA-3′ (shOPA3-2), and 5′-CAAGCCGCTTGCCAACCGT-3′ (shOPA3-3). To product lentiviruses, 2.8 μg of plasmid psPAX2 (Addgene, #12260), 1.6 μg of pMD2.G vector (Addgene, #12259) and 2 μg of shRNA plasmid were co-transfected into HEK293T cells (1.4 million cells) cultured in T-25 flasks using X-tremeGENE HP transfection reagent (Roche, Basel, Switzerland) according to manufacturer’s protocol. After 60 h, the medium containing viruses was collected and centrifuged for 10 min at 3000 rpm at 4 °C and then passed through a 0.45 µm filter. Cells were seeded in 6-well plate and infected with viruses and 8 µg/mL of polybrene-containing medium. Viruses were removed 24 h after infection and cells were selected with 1–2 μg/mL puromycin (Invivogen, Waltham, MA, USA) for 2 weeks. Stable transfectant cell lines were then obtained and characterized by immunoblotting for OPA3 protein levels.

### 4.3. Cell Transfection

Transfection was performed on cells at 50% confluence for 24 h with a 100 nM siRNA solution using Lipofectamine RNAiMax (ThermoFischer, Rockford, IL, USA) according to manufacturer’s protocol. Cells were then washed with PBS and replaced with DMEM for the indicated times. The sequences of siRNA for human OPA3 are: 5′-CCTATGGCGAAGCTGCTAT-3′ and 5′-CTATGGCGAAGCTGCTATA-3′.

### 4.4. Real-Time PCR

Total RNA was isolated using RNA-Quick Purification Kit (ESscience, Shanghai, China) according to the manufacturer’s instructions. RNA was reverse-transcribed using Primer Script RT reagent Kit with gDNA Eraser (Takara BIO INC, Kusatsu, Shiga, Japan). Real-time PCR was performed using the SYBR Premix Ex Taq RNAse H + kit (Takara), and analyzed using the Bio-Rad detection system (Bio-Rad, Hercules, CA, USA). The samples were first incubated 5 min at 95 °C, followed by 40 cycles of 10 s at 95 °C and 30 s at 60 °C. The results were calculated (formula: 2^−(Ct target-Ct EF1)^) and matched to the control samples. The primer sequences are listed in Supplementary Table S1, and were produced by Sangon Biotech (Shanghai, China).

### 4.5. Confocal Microscopy

Cells (1 × 10^5^) were plated on glass bottom microwell dishes (NEST, Jiangsu, China; #801002). The next day, culture medium was removed, cells were washed and stained with 100 nM MitoTracker red probe (Molecular Probes, Eugene, OR, USA) for 30 min. Fluorescence was observed by using an LSM880 confocal microscope (Zeiss, Oberkochen, Germany) equipped with a 63 × oil objective.

### 4.6. Immunoblotting

The procedures for protein sample preparation from cell cultures, protein quantification, immunoblotting, and data analyses were performed as previously described [23]. The following antibodies were used for immunoblotting analyses: β-actin (#ab6276), DRP1 (#ab56788), and OPA3 (#ab230205) were from Abcam (Cambridge, UK); phospho-DRP1 Ser616 (#3455), Vimentin (#5741), Slug (#9585), N-Cadherin (#13116), E-Cadherin (#3195) and snail (#3879) were from Cell Signaling Technology (Danvers, MA, USA); K-ras (#sc-30) was from Santa-Cruz biotechnology (Santa Cruz, CA, USA); and Tubulin (#BM1452) was from Boster Bio (Pleasonton, CA, USA). Protein bands were detected by chemiluminescence, using an ECL detection kit (Thermo Scientific). When appropriate, bands obtained via Western blot analysis were quantified, using the ImageJ software (http://rsb.info.nih.gov/ij/). Protein expression was normalized by β-actin or tubulin of the respective samples.

### 4.7. Immunohistochemistry

Human pancreatic cancer tissue microarray (Shanghai Outdo Biotech, China) was first dried at 58 °C for 1 h, dewaxed and rehydrated before epitope-retrieval by heating at 100 °C in 10 mM sodium-citrate (pH 6.0) for 4 min. The sections were cooled down to room temperature for 30 min. To eliminate the endogenous peroxidase and alkaline phosphatase activity in the tissue, the tissue sections were treated with 3% hydrogen peroxide for 20 min. The sections were then incubated with the individual primary antibody (OPA3, Proteintech, Rosemont, IL, USA; #15638-1-AP) diluted 1:50 overnight, followed by incubation with secondary anti-rabbit IgG antibody for 1 h. DAB (3,3’-diaminobenzidine) was then applied as a substrate to reveal the antigen. Hematoxylin was used for counterstaining. All other reagents were from ZSGB-Bio (Beijing, China). Scoring of the immuno-stained tissue sections was performed in a blind fashion, and recorded as score 0 (no target protein staining), score 1 (low staining), score 2 (intermediate staining), or score 3 (high staining). Results were quantified by multiplying the percentage of positive cells by the staining intensity scores (0–3), with a maximum score of 300 (3 × 100). The clinicopathological information of the patient samples is provided in Supplementary Table S2.

### 4.8. Measurement of Oxygen Consumption Rate (OCR) and Extracellular Acidification Rate (ECAR)

To estimate the rate of glycolysis and mitochondrial respiration, a Seahorse XFe/XF Analyzer (Agilent, Santa Clara, CA, USA) was used according to the manufacturer’s protocol. Briefly, 4 × 10^4^ cells/well were seeded in culture medium and the sensor cartridge was hydrated in Seahorse XF Calibrant buffer at 37 °C in incubator in absence of CO_2_ for 24 h. Then, culture medium was removed and replaced by Seahorse assay media. For the mitochondrial respiration test, the medium (pH 7.4) contained 1 mM pyruvate, 2 mM glutamine, and 10 mM glucose. The mitostress assay was performed by sequentially injecting 1 μM oligomycin, 1 μM carbonyl cyanide p-trifluoromethoxyphenylhydrazone (FCCP), and 0.5 μM rotenone/0.5 μM antimycin A. The cell number adjustment/normalization was performed using BCA.

### 4.9. Measure of Extracellular Lactate and Glucose

The concentrations of lactate and glucose were measured in culture medium using a SBA-90 Biosensor Analyzer (Biology Institute of Shandong Academy of Sciences, Jinan, China).

### 4.10. Measure of ATP Content

To measure ATP levels, cells (1 × 10^4^) were seeded in black 96-well plates and the ATP content was assessed using the bioluminescence CellTiter-Glo viability assay kit (Promega, Madison, WI, USA) according to the procedure provided by the manufacturer. Results are expressed as percent ATP levels of control (shCTL) cells.

### 4.11. Cell Proliferation Assays

Clonogenic assays were performed by seeding cells (500) in 6-well plates at a single cell density and allowed to grow for 14 days. Clonogenic survival was determined by staining colonies using crystal violet and data were expressed by the number of colonies.

For cell growth assay, 1 × 10^5^ cells were seeded in 6-well plates and counted every 24 h with an automated cell counter (Cellometer Auto T4; Nexcelom Bioscience, Lawrence, MA, USA).

### 4.12. Apoptosis

Panc-1 shRNA control or shRNA OPA3 cells were seeded into 12-well plates (4 × 10^5^). After incubating the cells for 72 h, cells were washed twice with cold PBS and then resuspended in Binding Buffer (BD Biosciences, San Jose, CA, USA) at a concentration of 1 × 10^6^ cells/mL. Cells were stained with PE-Annexin V and 7-AAD according to manufacturer’s instructions (PE Annexin V Apoptosis Detection Kit I; BD Biosciences; # BD #559763). Stained cells were analyzed using Beckman flow cytometer (Beckman Coulter, Miami, FL, USA).

### 4.13. Statistical Analyses

All experiments were performed separately at least three times. Student’s t-test was used to evaluate the statistical significance of the difference between two groups of samples with normal distributions. When there were more than two independent groups, ANOVA were used to compare the means of samples with normal distribution. Dunnett post hoc tests were performed when ANOVA was significant. When groups under different experimental conditions were compared, two-way ANOVA test was used. Statistical analyses were performed with GraphPad Prism software (San Diego, CA, USA). All tests were two-tailed, and a *p* value of 0.05 or less was considered as statistically significant.

## 5. Conclusions

Activation of oncogenic K-ras induces mitochondrial OPA3 expression, which is up-regulated in pancreatic cancer tissues. The main role of OPA3 appears to promote mitochondrial energy metabolism, and its up-regulation in K-ras-driven cancer is likely a mechanism to offset the negative impact of K-ras on mitochondria so that cellular energy homeostasis can be maintained. As such, OPA3 could be a potential target to kill K-ras-driven cancer cells.

## Supplementary Materials

The following are available online at https://www.mdpi.com/2072-6694/12/1/65/s1, Figure S1: Oncogenic K-ras activation induces OPA3 expression in T-Rex/K-ras^G12V^ cells, Figure S2: K-ras induces Drp-1 phosphorylation, Table S1: Oligonucleotides (Real-time PCR), Table S2: Baseline clinical characteristics of PDAC patients.
