# Supplementary material for: Oncogenic K-ras Induces Mitochondrial OPA3 Expression to Promote Energy Metabolism in Pancreatic Cancer Cells
Source: Cancers (Basel). 2019 Dec 25;12(1):65. doi: 10.3390/cancers12010065 (PMC7016999; doi:10.3390/cancers12010065)
Supplement: Supplementary file 1 [file cancers-12-00065-s001.pdf]

Supplementary material

Oncogenic K-ras Induces Mitochondrial OPA3 Expression to Promote Energy Metabolism in Pancreatic Cancer Cells

Ning Meng , Christophe Glorieux , Yanyu Zhang , Liyun Liang , Peiting Zeng , Wenhua Lu 1 and Peng Huang

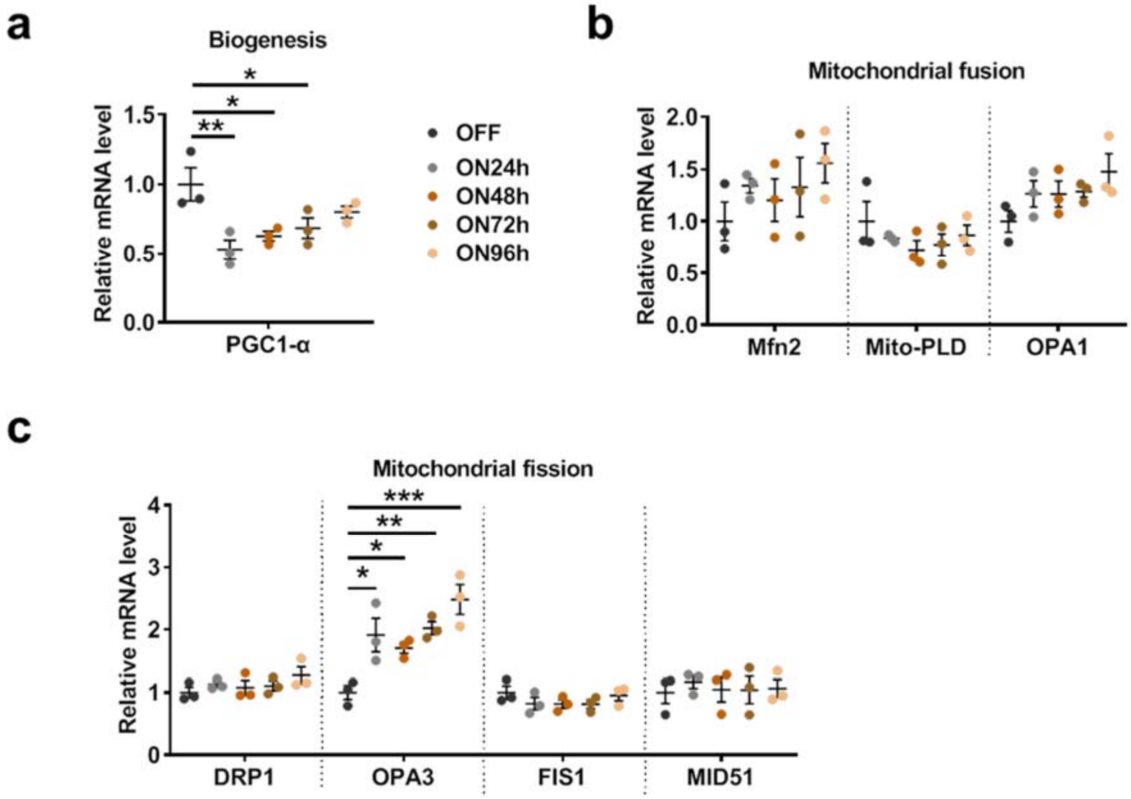

Figure S1. Oncogenic K-ras activation induces OPA3 expression in T-Rex/K-ras^{G12V} cells: Inducible K-ras^{G12V} cell line was incubated with doxycycline for various incubation times.The mRNA levels of (a) biogenesis; (b) mitochondrial fusion and (c) mitochondrial fission genes were measured by real-time PCR. Statistics: data are mean ± S.E.M. (n=3); one-way ANOVA followed by Dunnett post hoc test (compared to "OFF") for a-c. *P < 0.05, **P < 0.01, and ***P < 0.001. Abbreviations: Drp1: dynamin related protein 1; Fis1: mitochondrial fission 1 protein; Mfn2: mitofusin-2; MID51: mitochondrial dynamics proteins of 51 kDa; Mito-PLD: mitochondrial phospholipase D; OPA1: optic atrophy protein 1; OPA3: optic atrophy protein 3; PGC-1α: peroxisome proliferator-activated receptor gamma coactivator 1-alpha.

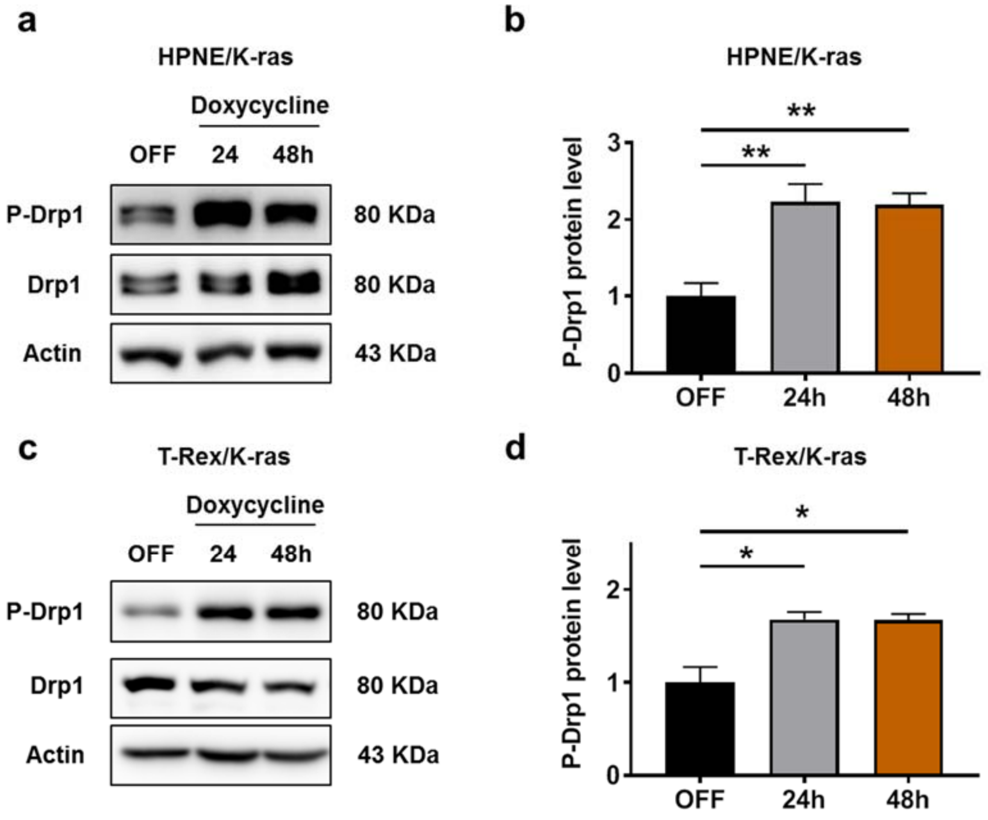

Figure S2. K-ras induces Drp-1 phosphorylation: (a-b) Inducible HPNE/K-ras^{G12D} cell line was incubated with doxycycline for various incubation times. Drp-1 and phospho-Drp1 (ser616) protein levels were analyzed by immunoblotting and quantified. (c-d) Inducible T-Rex/K-ras^{G12V} cell line was incubated with doxycycline for various incubation times. Drp-1 and phospho-Drp1 (ser616) protein levels were analyzed by immunoblotting and quantified. Statistics: data are mean ± S.E.M. (n=3); one-way ANOVA followed by Dunnett post hoc test (compared to "OFF") for b and d. *P < 0.05, **P < 0.01. Abbreviations: Drp1: dynamin related protein 1.

Table S1. Oligonucleotides (Real-time PCR)

Accession number	Forward	Reverse
NM_001017989.2	ggcgaagctgctatacttgg	gcattgaaacccatgatgc
NM_001402.5	gcttcactgctcaggtgat	gccgtgtggcaatccaat
NM_033540.2	atgctcaaagggtgctccta	agatgtaacggacgccaatc
NM_014874.3	catgggcattcttgttgttg	tggagccagtgtagctgatg
NM_178836.3	aagaatttgagcgcatctgg	gttctgtgccatgaaagcaa
NM_015560.2	ggattgtgcctgacattgtg	ggattgtgcctgacattgtg
NM_004331.2	agcagggaccatagctctca	tcatggctccacttttcctc
NM_032409.2	gaagccaccatgcctacatt	agctcctggctcattgtgtt
NM_012062.4	acccggagacctctcattc	tgacaacgttgggtgaaaaa
NM_016068.2	ttcagtctgagaaggcagca	acggccaggtagaagacgta
NM_019008.5	ccacatccaactcattgtgc	agccagccactaccttctca
NM_001330751.1	caccagccaacactcagcta	gtgtgaggagggtcatcgtt

Table S2. Baseline clinical characteristics of the PDAC patient samples.

**PDAC**	**N**	**%**
Total	65	100
Score 0-150	13	20
Score > 150	52	80
(Max = 300)		
**T stage**		
T1	4	6.1
T2	56	86.1
T3	5	7.8
**N stage**		
Unknown	4	6.1
N0	41	63.1
N1	20	30.8
**M stage**		
M0	61	93.9
M1	4	6.1

**Score 0-150**	**N**	**%**
Total	13	100
**T stage**		
T1	3	23.1
T2	10	76.9
T3	0	0
**N stage**		
Unknown	1	7.7
N0	11	84.6
N1	1	7.7
**M stage**		
M0	13	100
M1	0	0

**Score > 150**	**N**	**%**
Total	52	100
**T stage**		
T1	1	1.9
T2	46	88.5
T3	5	9.6
**N stage**		
Unknown	3	5.8
N0	30	57.7
N1	19	36.5
**M stage**		
M0	48	92.3
M1	4	7.7

© 2019 by the authors. Licensee MDPI, Basel, Switzerland. This article is an open access article distributed under the terms and conditions of the Creative Commons Attribution (CC BY) license (http://creativecommons.org/licenses/by/4.0/).
